# Endovascular embolization for primary racemose hemangioma of the bronchial artery supplied by multiple systemic arteries: A rare case report

**DOI:** 10.1097/MD.0000000000046176

**Published:** 2026-01-02

**Authors:** Hao Lin, Qunfeng Yan, Yunyu Lin, Dafeng Hong, Chao Qiu, Sen Jiang

**Affiliations:** a Department of Respiratory Medicine, Ninghai First Hospital, Ningbo, Zhejiang, China; b Department of Radiology Department, Ninghai First Hospital, Ningbo, Zhejiang, China; c Department of Radiology, Shanghai Pulmonary Hospital, School of Medicine, Tongji University, Shanghai, China.

**Keywords:** bronchial artery aneurysm, case report, embolization, racemose hemangioma of the bronchial artery

## Abstract

**Rationale:**

Primary racemose hemangioma of the bronchial artery (RHBA) is a rare congenital vascular malformation, typically confined to the bronchial artery. Concomitant vascular supply originating from multiple non-bronchial systemic arteries (NBSAs) is exceedingly rare and significantly complicates treatment procedures.

**Patient concerns:**

We report an asymptomatic 66-year-old woman in whom incidental imaging revealed RHBA sustained by a complicated network of multiple systemic feeding arteries.

**Diagnoses:**

Chest computed tomography angiography and confirmatory selective arteriography revealed 6 markedly dilated, tortuous mediastinal arteries extending from the right subclavian artery to the abdominal aorta, terminating in multiple aneurysms clustered at the right hilum.

**Interventions:**

Successful endovascular embolization was achieved via super-selective catheterization and microcoils deployment. Six-month follow-up confirmed durable occlusion without recurrence.

**Lessons:**

RHBA with multi-source NBSAs involvement is rare. During interventional procedure, it must carefully consider the influence of diverse arterial blood flow on distal ectopic embolization, and omission of any feeding NBSAs can precipitate RHBA recurrence. In this case, carefully super-selective catheterization combined with microcoils deployment offered a safe, effective therapeutic option.

## 1. Introduction

Primary racemose hemangioma of the bronchial artery (RHBA) is a rare congenital vascular malformation charactered as dilated and tortuous bronchial artery (BA), and occasionally complicated by aneurysms.^[1,2]^ RHBA can also have abnormal fistulas between pulmonary artery (PA) or pulmonary vein, but normally does not have connections with other non-bronchial systemic arteries (NBSA). Given the fatal hemorrhage of aneurysm rupture, RHBA with aneurysm should be treated once detected,^[1,3–6]^ and endovascular treatments are the first-line therapeutic strategy.^[7–9]^ In addition, the previous studies have reported the involvement of multiple NBSAs in the vascular supply of RHBA could significantly complicate interventional strategy.^[10]^ In the present report, we reported an exceedingly rare case of RHBA supplied by 6 systemic arteries, successfully embolized by metallic microcoils.

## 2. Case presentation

**Figure 1.**
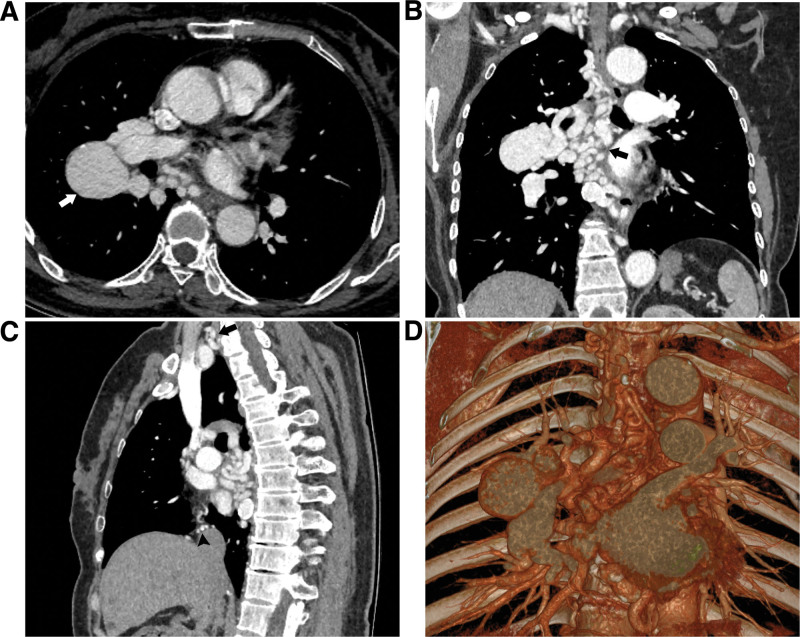
(A) The right BA is markedly dilated and tortuous, with multiple aneurysms, largest located at the right hilum (white arrow). (B), (C) Multiplanar reconstruction images reveal multiple dilated and serpiginous systemic arteries that filled the mediastinum. (D) Three-dimensional reconstruction image clearly shows multiple communicating systemic arteries within the mediastinum converges and drains into the largest aneurysm.

This retrospective study has been approved by the ethics committee of Shanghai Pulmonary Hospital, and the ethics committee of Shanghai Pulmonary Hospital (K24-522) declared the informed consent can be waived because of its retrospective nature. The patient provided written informed consent for the publication of medical data. A 66-year-old woman presented with a mass shadow in the right hilum of the lung on a regular health checkup chest X-ray. She had no respiratory symptoms such as cough, sputum production, or chest pain, and no history of chronic bronchitis, bronchiectasis, or other respiratory diseases. She had a history of hypercholesterolemia and hypertension. Chest CT scan showed no obvious abnormalities in both lungs. Chest computed tomography angiography images demonstrated significant tortuosity and dilation of multiple systemic arteries, with multiple aneurysms in the right hilum and mediastinum. The largest aneurysm, measuring 37 × 40 × 45 mm, was located in the right hilum. Three-dimensional reconstruction images revealed that the right BA, right costocervical trunk, right thyrocervical trunk, right internal thoracic artery, esophageal artery and right inferior phrenic artery were all abnormally tortuous and dilated, with multiple aneurysms locally (Fig. 1). The 6 arteries eventually drained into the largest aneurysm.

**Figure 2.**
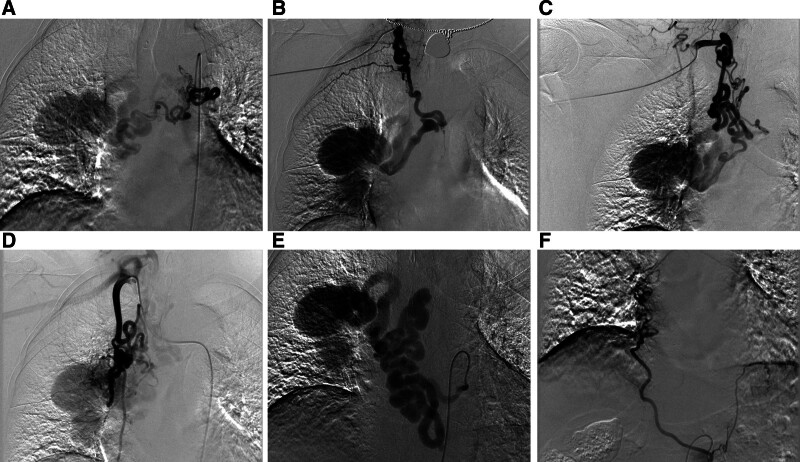
(A) The orthotopic right BA arising from the thoracic aorta. (B) The ectopic right BA originating from the right costocervical trunk. (C) The other ectopic right BA originating from the right thyrocervical trunk. (D) One dilated and tortuous branch of the right internal thoracic artery drains into the aneurysm. (E) One markedly dilated and tortuous esophageal artery drains into the aneurysm. (F) The distal segment of the right inferior phrenic artery, arising from the abdominal aorta, drains into the aneurysm.

Endovascular treatment was then performed under local anesthesia. Right femoral and radial arterial access was obtained via the modified seldinger technique. Selective angiography showed no direct connection between the left BA and the RHBA. Six systemic arteries converged to form the RHBA: one orthotopic right BA arising from the thoracic aorta, 2 aberrant right bronchial arteries arising from the right costocervical and thyrocervical trunks, respectively, one esophageal artery, one right inferior phrenic artery, and one right internal thoracic artery. Angiography revealed the presence of multiple aneurysms in the right hilar and mediastinal regions, characterized by rapid blood flow. A direct, high-flow fistula between the right PA trunk and the largest aneurysm was also revealed. Notably, 3 right BAs converged distally and drained into this largest aneurysm, and 3 other systemic arteries independently drained into the largest aneurysm. Pulmonary angiography via the right femoral vein access confirmed aneurysmal dilatation of the main PA trunk and a high-flow shunt into the largest systemic aneurysm (Fig. 2).

**Figure 3.**
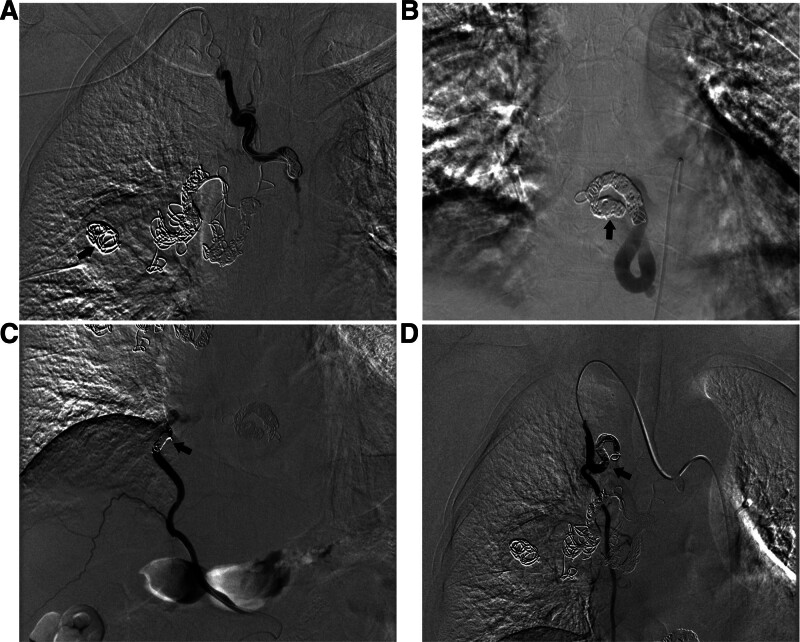
(A) The ectopic right BA, arising from the right costocervical trunk, was completely occluded with micro coils; the 2 coils that had migrated distally during embolization are marked by black arrow. (B) The esophageal artery was completely occluded, as denoted by the black arrow. (C) The distal segment of inferior phrenic artery was also embolized with micro coils. (D) The branch of the internal thoracic artery draining into the RHBA were also completely embolized (black arrow).

One 1.98-F microcatheter navigated over a 0.014-inch micro-guide wire achieved super-selective catheterization of the ectopic right BA (arising from the right costocervical trunk) up to the confluence of 3 BAs. The catheterized BA was occluded with a total of 30 coils: 3 Guglielmi detachable coils (GDC, 16-3D-30 cm, 14-1D-30 cm, 14-3D-30 cm) followed by 27 micro coils (thirteen 18-14-10-NESTER, 3 18-14-8-NESTER, 4 18-14-6-NESTER, 2 18S-8/4-TORNADO, and 5 18S-10/4-TORNADO) (Cook Medical) deployed in tight succession. Unexpectedly, the first 2 18-14-10-NESTER coils migrated distally due to the competing blood flow of other 2 converged BAs. After super-selective catheterization, the esophageal artery was occluded with thirteen micro coils: 5 18-14-10-NESTER, one 18-14-8-NESTER, 3 18-14-6-NESTER, one 18S-8/4-TORNADO, and 3 18S-10/4-TORNADO. The inferior phrenic artery was then embolized with 3 micro coils (one 18-14-8-NESTER, one 18-14-6-NESTER, one 18S-7/3-TORNADO) following the same procedure. Finally, the internal thoracic artery was occluded with 7 micro coils: 2 18-14-8-NESTER, 3 18-14-6-NESTER, one 18S-8/4-TORNADO, and one 18S-7/3-TORNADO. Post-embolization angiography confirmed complete occlusion of the RHBA (Fig. 3). The patient was transferred to the ward in stable condition.

**Figure 4.**
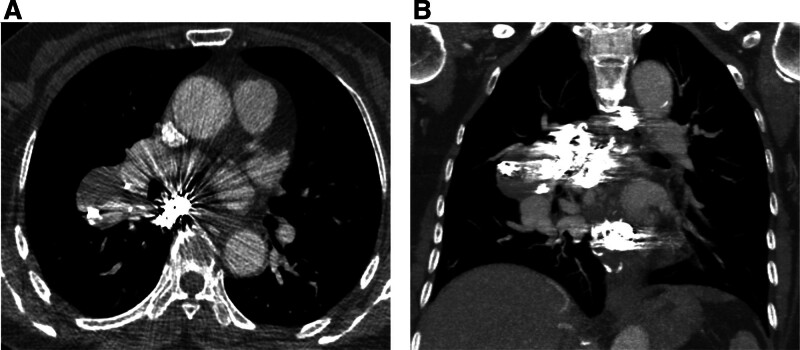
(A) Enhanced CT images demonstrate complete occlusion of the BA, reduction in the size of the largest aneurysm, and formation of an intracavitary thrombus. (B) Coronal CT imaging reveals post-embolization changes in multiple mediastinal arteries, with no evidence of coil migration.

The patient received oxygen therapy (3L/min) for one day following the embolization procedure and did not experience any discomfort. One-month post-procedural aortic angiography revealed no evidence of RHBA recurrence. The patient was subsequently followed up for 6 months, during which enhanced CT images demonstrated no recanalization of the occluded RHBA (Fig. 4).

## 3. Discussion

RHBA was first described by Babo et al in 1976 and had since been widely recognized in Japan.^[11,12]^ RHBA is characterized by significant bending, dilation, and tortuosity of BAs, occasionally complicated by aneurysms. Additionally, RHBA often involves abnormal fistula between the BA and PA. Repeated hemoptysis is the most prominent symptom. While few patients may have no obvious symptoms.^[13,14]^ RHBA is classified into 2 types: primary and secondary. Primary RHBA is mainly attributed to congenital vascular malformations, but the exact genetic mechanisms remain to be fully elucidated. Secondary RHBA typically arises from chronic pulmonary inflammation, such as bronchiectasis or tuberculosis.^[6,13]^ In the present case, the patient displayed no obvious pulmonary abnormalities, signs of inflammation, or relevant clinical symptoms. RHBA was detected incidentally during a routine physical examination, leading to the diagnosis of primary RHBA.

RHBA primarily affects the BA, and the involvement of multiple NBSAs in RHBA is uncommon. However, in this case, multiple NBSAs were also involved, including the right subclavian trunk, right thyrocervical trunk, right internal thoracic artery, right esophageal artery, and the right phrenic artery. Although a limited number of previous studies have reported the participation of NBSAs in the vascular supply of RHBA, the simultaneous involvement of 5 NBSAs originating from such a wide anatomical range, from the subclavian artery to the abdominal aorta, is exceptionally rare.^[10,15,16]^ The presence of 5 supplying NBSAs substantially increased the complexity of this RHBA case, leading to a more extensive lesion distribution and more intricate hemodynamic patterns due to multiple arterial flow. This multifaceted vascular involvement presents significant challenges for clinical management and interventional strategies.^[10]^

Given the potentially life-threatening hemorrhage risk from ruptured RHBA with BAA, treatment is recommended regardless of the presence of clinical symptoms.^[5,9,17]^ Therapeutic candidates include thoracoscopic BA resection, lobectomy, and endovascular therapy.^[16,18,19]^ Among them, endovascular therapy has recently emerged as the first-line treatment.^[14,19]^ In this case, the RHBA involved multiple arteries with different origins, making surgical ligation challenging and invasive. Additionally, it might require right pneumonectomy due to extensive lesions, causing significant pulmonary function loss. Considering all these factors, endovascular treatment was determined to be the most feasible strategy in our case.

Based on previous studies, the available embolic materials for RHBA include aortic covered stents, metallic coils, vascular plugs, and peripheral embolic agents, such as N-butyl-2-cyanoacrylate (NBCA) and particulate embolic agents.^[10,15,20]^ Covered stents can effectively isolate blood flow from the aorta to RHBA, but they are not suitable for cases involving multiple NBSAs with dispersed origins due to the risks of paraplegia, endo-leak, and incomplete isolation.^[8]^ Peripheral embolic agents have good fluidity, which allows them to pass through tortuous vessels to achieve distal embolization.^[21]^ However, when there is a fistula between RHBA and PA, low-concentration NBCA and particulate embolic agents could result in severe ectopic embolism.^[12,14]^ While high-concentration NBCA could cause incomplete embolization and easily lead to vascular recanalization and recurrence.^[6,11]^ Therefore, in this case, super-selective catheterization using a microcatheter and subsequent coil embolization were performed.

Previous studies have shown that distal embolization could reduce recanalization and recurrence in RHBA.^[11,22]^ In this case, the RHBA had multiple feeding arteries with complex anatomy, including tortuosity and dilation, which complicated distal embolization: tortuous and dilated vessels hindered catheter navigation to the distal segment of the targeted artery; the involvement of multiple NBSAs increased the complexity of treatment, and the omission of any one artery can lead to incomplete embolization; the confluence of multiple feeding arteries resulted in increased blood flow velocity, which could cause embolic material migration.^[6]^ To address these challenges, we super-selective catheterized the right costocervical trunk to the confluence of feeding arteries and deployed multiple detachable coils as anchors. However, some coils placed at confluence segment migrated into the largest BAA due to high-velocity blood flow. Successful embolization was achieved only after deploying several oversized coils. This underscores the necessity of considering hemodynamic effects when treating RHBA with multiple feeding arteries and large fistulas between the BAA and PA to avoid complications such as ectopic embolization.^[19]^

## 4. Conclusion

Primary RHBA involving multiple NBSAs with dispersed origins was rare and complex. Treatment must fully consider the feeding arteries’ origin, number, and anatomy, along with intricate hemodynamic pattern. Super-selective catheterization combined with appropriate micro-coil embolization is a safe and effective endovascular interventional therapy.

## Acknowledgments

We thank the patient for granting permission to share the medical data. We thank Angela Morben, DVM, ELS, from Liwen Bianji, Edanz Editing China (www.liwenbianji.cn/ac), for editing the English text of a draft of this manuscript.

## Author contributions

**Conceptualization:** Hao Lin, Sen Jiang.

**Data curation:** Hao Lin, Sen Jiang.

**Formal analysis:** Hao Lin, Sen Jiang.

**Investigation:** Hao Lin, Qunfeng Yan, Sen Jiang.

**Methodology:** Hao Lin, Qunfeng Yan, Dafeng Hong, Sen Jiang.

**Writing – original draft:** Hao Lin, Sen Jiang.

**Supervision:** Qunfeng Yan, Dafeng Hong, Sen Jiang.

**Writing – review & editing:** Qunfeng Yan, Yunyu Lin, Dafeng Hong, Chao Qiu, Sen Jiang.

**Validation:** Yunyu Lin, Sen Jiang.

**Visualization:** Yunyu Lin, Dafeng Hong, Chao Qiu, Sen Jiang.

**Software:** Dafeng Hong.
